# Enrichment of Grapes with Zinc-Efficiency of Foliar Fertilization with ZnSO_4_ and ZnO and Implications on Winemaking

**DOI:** 10.3390/plants11111399

**Published:** 2022-05-25

**Authors:** Diana Daccak, Fernando C. Lidon, Cláudia Campos Pessoa, Inês Carmo Luís, Ana Rita F. Coelho, Ana Coelho Marques, José C. Ramalho, Maria José Silva, Ana Paula Rodrigues, Mauro Guerra, Roberta G. Leitão, Paula Scotti Campos, Isabel P. Pais, José N. Semedo, Maria Manuela Silva, José Carlos Kullberg, Maria Brito, Carlos Galhano, Paulo Legoinha, Maria Fernanda Pessoa, Manuela Simões, Fernando H. Reboredo

**Affiliations:** 1 Earth Sciences Department, Faculdade de Ciências e Tecnologia, Universidade Nova de Lisboa, Campus da Caparica, 2829-516 Caparica, Portugal; fjl@fct.unl.pt (F.C.L.); c.pessoa@campus.fct.unl.pt (C.C.P.); idc.rodrigues@campus.fct.unl.pt (I.C.L.); arf.coelho@campus.fct.unl.pt (A.R.F.C.); amc.marques@campus.fct.unl.pt (A.C.M.); jck@fct.unl.pt (J.C.K.); mgb@fct.unl.pt (M.B.); acag@fct.unl.pt (C.G.); pal@fct.unl.pt (P.L.); mfgp@fct.unl.pt (M.F.P.); mmsr@fct.unl.pt (M.S.); fhr@fct.unl.pt (F.H.R.); 2 GeoBioTec Research Center, Faculdade de Ciências e Tecnologia, Universidade Nova de Lisboa, Campus da Caparica, 2829-516 Caparica, Portugal; cochichor@mail.telepac.pt (J.C.R.); mjsilva@isa.ulisboa.pt (M.J.S.); paula.scotti@iniav.pt (P.S.C.); isabel.pais@iniav.pt (I.P.P.); jose.semedo@iniav.pt (J.N.S.); abreusilva.manuela@gmail.com (M.M.S.); 3 PlantStress & Biodiversity Laboratory, Centro de Estudos Florestais (CEF), Instituto Superior Agronomia (ISA), Universidade de Lisboa (ULisboa), Quinta do Marquês, Av. República, 2784-505, Oeiras and Tapada da Ajuda, 1349-017 Lisboa, Portugal; anadr@isa.ulisboa.pt; 4 LIBPhys, Physics Department, Faculdade de Ciências e Tecnologia, Universidade Nova de Lisboa, Campus da Caparica, 2829-516 Caparica, Portugal; mguerra@fct.unl.pt (M.G.); rg.leitao@fct.unl.pt (R.G.L.); 5 Instituto Nacional de Investigação Agrária e Veterinária, I.P. (INIAV), Avenida da República, Quinta do Marquês, 2780-157 Oeiras, Portugal; 6 Escola Superior de Educação Almeida Garrett (ESEAG-COFAC), Avenida do Campo Grande 376, 1749-024 Lisboa, Portugal

**Keywords:** agronomic enrichment with zinc, Castelão, Moscatel, *Vitis vinifera*

## Abstract

Grapes and wine are widely consumed in the world, yet their mineral content can be influenced by many factors such as the mineral composition of soils, viticulture practices and environmental conditions. In this context, considering the importance of Zn in the human physiology, the enrichment of Moscatel and Castelão grapes (white and red variety, respectively) with this nutrient prompted this study; further assessment of tissue deposition and some implications for wine production. Using two foliar fertilizers (ZnO or ZnSO_4_, at 150, 450 and 900 g ha^−1^), decreases in net photosynthesis and stomatal conductance occurred in both varieties, suggesting that the physiological threshold of Zn toxicity was reached without visible symptoms. Following foliar spraying with both fertilizers, the content of Zn in leaves of the Castelão and Moscatel varieties showed higher values in all treatments relative to the control. Moreover, in grapes this tendency occurred only in Castelão. Concerning Cu, Fe, Ca, K, S and P, some significant differences also happened in leaves and grapes among treatments. At harvest, the indexes of Zn enrichment in grapes increased between 2.14- and 8.38-fold and between 1.02- and 1.44-fold in Castelão and Moscatel varieties, respectively. Zinc in the dried skin of Castelão only increased with ZnO and ZnSO_4_ sprayed at 900 g ha^−1^ (ca. 2.71- and 1.5-fold relative to the control, respectively), but in Moscatel a clear accumulation trend could not be found. The dry weight of grapes ranged (in %) between 16 and 23 (but did not vary significantly among treatments of each variety or in each treatment between varieties), and total soluble solids (e.g., mainly soluble sugars and proteins) and color parameters showed some significant variations. Through winemaking, the contents of Zn increased in both varieties (1.34- and 3.57-fold, in Castelão and Moscatel, respectively) and in all treatments, although non-significantly in Castelão. It is concluded that, to increase the contents of Zn in grapes without reaching the threshold of toxicity, ZnO or ZnSO_4_ can be used for foliar spraying of Castelão and Moscatel varieties until 900 g ha^−1^ and that winemaking augments the level of this nutrient.

## 1. Introduction

Zinc, the 23rd most abundant element on earth, is a transition metal with the atomic number 30 and is redox-stable under physiological conditions because of a complete d-shell of electrons [1,2]. Its divalent cation (Zn^2+^) has an affinity for negatively charged hydroxyl and thiol groups, and readily forms complexes with aminoacids, peptides, proteins and nucleotides. In this context, at cellular and tissue levels, zinc’s multiple functions include catalytic, structural and regulatory roles. Thus, Zn binding sites exist in a large number of proteins, membrane lipids and nucleic acid molecules. The largest class of Zn-binding proteins in organisms is the Zn finger domain containing proteins, which can regulate transcription directly through effects on DNA/RNA-binding, and also through site-specific modifications, regulation of the chromatin structure, RNA metabolism and protein–protein interactions [3,4,5]. Typically, Zn is the second most abundant transition metal in organisms and the only metal represented in all six enzyme classes (Enzyme Commission number, EC 1–6: oxidoreductases, transferases, hydrolases, lyases, isomerases, ligases) [6].

Besides the structural and metabolic relevance of Zn at a cellular level, from a nutritional point of view, Zn is a fundamental trace element and one of the most abundant in the human body (i.e., its average content in adults ranges between 1.5–2.5 g) [5,7,8]. Considering the Zn linkage to the major biochemical pathways, namely, gene regulation and cellular division, severe Zn deficiency has adverse consequences, namely, depressed growth and genital development, immune and cognitive dysfunctions, diarrhea and reproductive teratogenesis [9,10,11,12]. If the human diet is low in protein, or if high intakes of inhibitors of zinc absorption (such as phytate) occur, or even if absorption is suboptimal in the distal duodenum and proximal jejunum of the small intestine, chronic mild or moderate zinc insufficiency can develop [9]. Indeed, in the human genome about 10% of proteins (i.e., 2800) potentially bind Zn [13], with hundreds more involved in Zn transport and trafficking [14]. Nevertheless, within a typical range of diets, small increases in consumed Zn substantially increase the total amount of this nutrient’s absorption in a non-linear manner, consistent with a saturable response [9]. In this context, although the recommended daily intake of Zn depends on several factors such as age, sex, weight and phytate content in the diet, 11 and 8 mg for adult males and females, respectively, have been indicated [7,9].

To surpass nutrient deficiency in the human diet, the application of agronomic workflows to increase the amount of a nutrient in the edible part of food crops, by application of fertilizers through soil or foliar spraying, is receiving increasing attention [15,16,17,18,19,20]. Among these approaches, Zn fertilization is a short-term and effective strategy [15], yet its absorption greatly depends on soil pH, organic matter concentration, antagonistic cations (especially in calcareous soils), type of Zn complex and amount of foliar spraying. In fact, Zn enrichment through soil fertilization involves many physiological steps including Zn uptake and its root-to-shoot translocation and remobilization [18,21,22]. Zn uptake is highly dependent on soil pH (i.e., being acquired, in a lesser extent, through mechanisms of mass flow and predominantly by diffusion) and follows apoplast and symplast pathways. Accumulation occurs in exchangeable forms in the apoplast (adsorbed to the negatively charged pectin matrix of the cell wall), as a labile form (related to nutrients located in the cytoplasm) and as a nonlabile form (as a nutrient allocated to the vacuole and not translocated in the plant) [23]. The efficiency of Zn uptake is also defined by the product of root Zn concentration and root dry weight, a process determined by the root surface area and root length density [22,24,25]. Although it depends on plant genotypes, translocation of Zn from root to shoot is affected by many factors, namely, by optimized levels of N [26], increased P application [27] and low levels of Zn in the subtract of the cultures that stimulates the translocation rates to shoots and remobilization to edible parts [21,25,28,29].

Through leaves, plants can also absorb different nutrients and export them within the stem via phloem or xylem [30,31], which implies that the foliar spray of micronutrients would, theoretically, determine how an applied nutrient can be reallocated from leaves to the growing tissues [32]. Indeed, plant growth, and even survival of new and young organs, is dependent on the remobilization of mineral nutrients in a limited nutrient availability. Once in the phloem, Zn mobility is intermediate, meaning that it is immobile or relatively mobile, depending on phenology and storage.

Although Zn enrichment occurs when its contents exceed the level of sufficiency within a crop plant, oversupply can have negative metabolic and structural consequences at cellular and tissue levels [33]. Indeed, a deficiency or excess of Zn can negatively affect water tensions, creating ionic unbalance, and inhibit many physiological processes, namely, the synthesis of protein and carbohydrates, auxin biosynthesis, cellular division, membrane structural integrity, photosynthesis and seed germination, due to its role as a cofactor for many enzymes [33,34]. Besides, protein-binding Zn also is central in a plant’s metabolism. For instance, using *Arabidopsis thaliana* as a reference biological system, 2367 proteins (implicating 181 gene families) were identified as Zn-related, which is the largest group assigned to transcription regulator activity and binding functional subcategories [35]. On the other hand, if the threshold of toxicity for Zn is not reached, in some crops and fruit trees the Zn content and yield after foliar application increase [10] and can even ameliorate toxicity triggered by other minerals [36]. For instance, foliar application of Zn in wheat increases the number of grains per spike, seed yield and Zn grain concentration [37]. At metabolic levels, foliar application with Zn also increases the levels of relative water content, total chlorophyll content and activities of antioxidant enzymes [38,39]. Accordingly, the effectiveness of natural enrichment with Zn must consider the specific requirements of plant genotypes and the timing of a micronutrient foliar spray at specific and critical stages during the life cycle [40,41,42,43,44].

Considering the importance of grapes and winemaking (vines were grown on 3.2 million hectares in the EU, representing about 45% of the world’s total area under vines), the efficiency of foliar fertilization with ZnSO_4_ and ZnO in vineyards of two contrasting varieties (Castelão and Moscatel) of *Vitis vinifera* L. for the enrichment of grapes with Zn prompted this study; furthermore, this study assessed its tissue deposition in grapes and the implications on winemaking.

## 2. Results

### 2.1. Fields Characteristics for Agronomic Enrichment with Zinc

**Figure 1:**
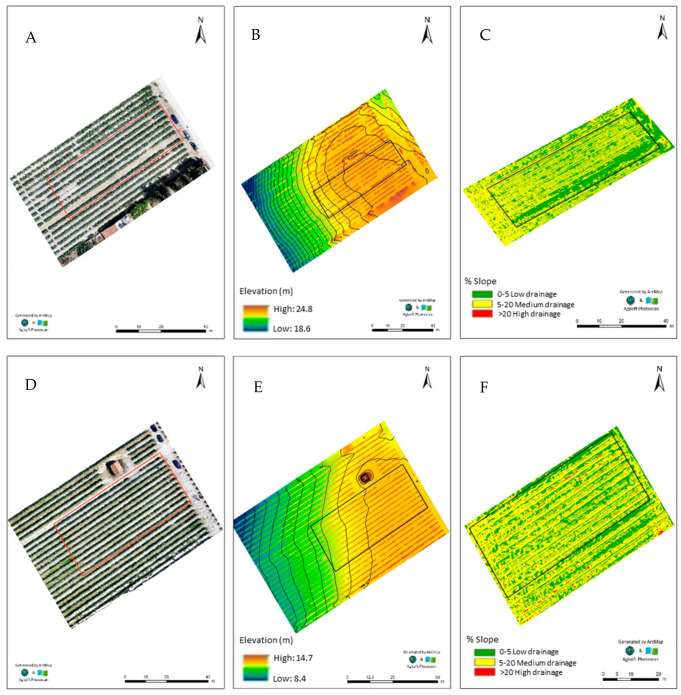
Orthophotomaps of the vineyards: Lagameças–Cv. Castelão (**A**–**C**) and Lau Novo–Cv. Moscatel (**D**–**F**). Indication (in red) of limits of the two fields (**A**,**D**); digital elevation model of the fields (**B**,**E**); digital map of slopes of the fields (**C**,**F**); information collected before flowering and enrichment treatments (1 August for both fields).

The vineyards of both varieties were almost flat with a very slight inclination, revealing a maximum variation of 0.80 m in Lagameças and about 1.10 m in Lau Novo (Figure 1). As the morphology strongly affects the drainage of surface water, the slopes of the field were calculated (Figure 1C,F; Table 1) and found that in Lagameças about 50% of the area can promote the accumulation and infiltration of surface water, whereas the remaining 50% corresponds to the aptitude for surface drainage (Figure 1C; Table 1). In the vineyard of Lau Novo (Figure 1C,F; Table 1) only 1/3 of the field has suitable conditions for surface water infiltration.

Comparatively to the vineyard of Lau Novo, the average values of pH, electrical conductivity and organic matter in the soils were found to be significantly higher in the vineyard of Lagameças (Table 1), indicating a higher salt content and, therefore, requiring more energy expenditure for water absorption by plants’ roots. Comparatively to Lau Novo, the soil of Lagameças revealed significantly higher average contents of Ca, Fe and Zn, and lower amounts of K and P (Table 1). Moreover, the average concentrations of Mg and S did not vary significantly in the soils of both vineyards (Table 1).

The irrigation water of Lau Novo (Table 1) was from an underground origin, with a predominance of sodium chloride sulfate and with intermediate salinity (concentration of salts evaluated, in terms of electrical conductivity, between 250 and 750 μS/cm at 20 °C); the irrigation water belongs to class C2S1, with SAR index 1.52.

### 2.2. Physiological Monitoring of Photoassimilates during Zinc Enrichment

**Figure 2:**
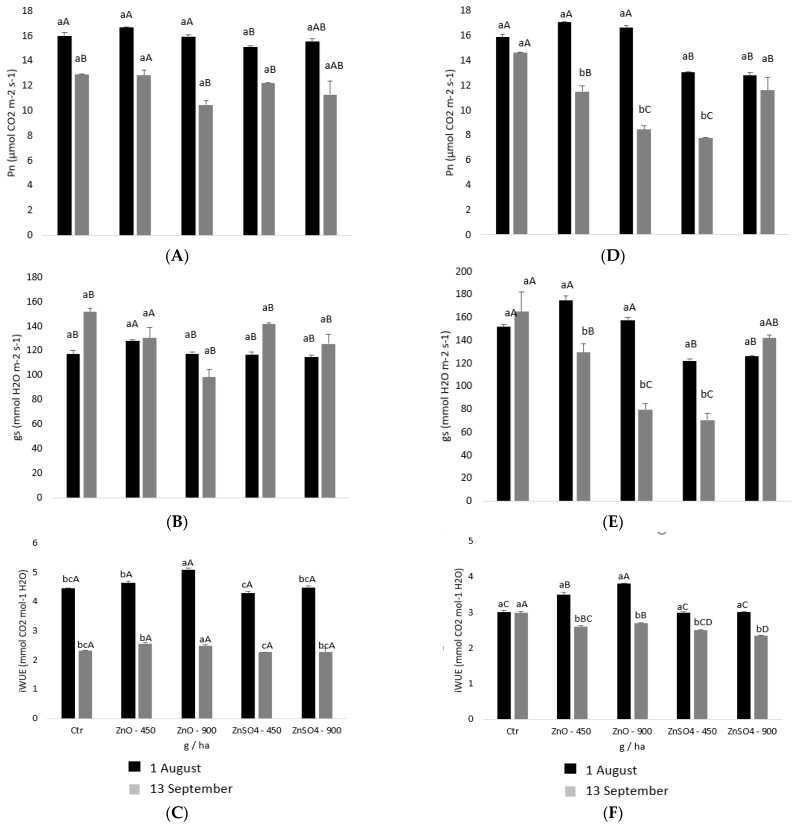
Average ± SE of leaf gas exchange parameters, net photosynthesis (Pn) and stomatal conductance to water vapor (gs), as well as variation in the instantaneous water use efficiency (iWUE = Pn/E) in leaves of *Vitis vinifera* of varieties Castelão (**A**–**C**) and Moscatel (**D**–**F**), after the third leaf spraying on 1 August (first assessment) and 13 September 2018 (second assessment) with ZnO and ZnSO_4_ at different concentrations. For all parameters, the mean value ± SE (*n* = 6) is succeeded by different letters indicating significant differences between testing parameters for the different treatments (a, b, c), or between different assessments in the same treatment (A, B, C, D) (statistical analysis using the two-way ANOVA test, *p* < 0.05).

The application of Zn fertilizers at non-toxic concentrations improves photochemical reactions in thylakoid membranes (ensuring membrane integrity), improves electron transport through the photosystem II and increases the photosynthetic rates and chlorophyll content, which determines an increase in fruit yield and quality as observed for kinnow mandarins, sweet oranges and grapes [45]. Based on this background, Pn, gs and iWUE were used for monitoring the potential effects of leaf spraying Castelão and Moscatel varieties at different concentrations with ZnO and ZnSO_4_. It was found that after three leaf sprayings (1 August), the Pn of Castelão treated with ZnO did not vary significantly among treatments, but pulverization with ZnSO_4_ (450 and 900 g ha^−1^) revealed about a 20% decrease (Figure 2A). After the 2nd assessment (13 September), both Zn chemical forms caused a decrease in Pn, with maximum reductions of 42% and 47% (i.e., through the application of 900 g ha^−1^ ZnO and 450 g ha^−1^ ZnSO_4_, respectively, and about 21% in the remaining treatments) due to variations of gs (Figure 2B). Indeed, gs showed reductions with the application of ZnSO_4_ on both assessment dates and, after the 2nd assessment, the application of ZnO also showed maximum decreases (52% with 900 g ha^−1^ ZnO and 57% with 450 g ha^−1^ ZnSO_4_). The combination of Pn and E results led to an iWUE reduction between 10% and 22% on the 2nd assessment (Figure 2C).

Through pulverization with both chemical forms, Zn and Pn, the Moscatel did not vary significantly after the first assessment, but following the second assessment decreases of 13% and 19% were found with the application of 900 g/ha (Figure 2D). These effects might be linked to the impact of gs only in the second assessment, which showed reductions of 17% and 37% in the same concentrations (900 g ha^−1^) of ZnSO_4_ and ZnO, respectively (Figure 2E). Contrary to Castelão, iWUE of all treatments in cv. Moscatel showed values close to the respective control (Figure 2F).

### 2.3. Nutrient Contents in Leaves and Grapes during Zinc Enrichment

After the second foliar application with ZnO or ZnSO_4_, the contents of nutrients in the leaves and grapes of Castelão and Moscatel showed (Table 2) some significant differences among treatments. Comparatively to the control, Zn content showed higher values in all treatments sprayed with both Zn chemical fertilizers (except in the grapes of Moscatel). The maximum content of Zn in leaves and grapes were obtain in both varieties with an application of 900 g ha^−1^ ZnO, but spraying with increasing concentrations of both fertilizers also augmented the content of Zn (except in the leaves and grapes of Castelão treated with 450 g ha^−1^ ZnSO_4_ and in the grapes of Moscatel treated with 900 g ha^−1^ ZnSO_4_).

In the leaves of both varieties, minimum contents of Ca were found in the control, whereas maximum values were found in Castelão and Moscatel treated with 900 g ha^−1^ of ZnO and ZnSO_4_, respectively (Table 2). Moreover, Ca accumulation in leaves only increased progressively in Moscatel treated with increasing amounts of ZnSO_4_ (Table 2). In the grapes of Castelão and Moscatel, a clear trend for Ca accumulation was not found when increasing concentrations of both fertilizers were applied.

The levels of Cu and K in the leaves of Castelão revealed minimum values with the highest treatment of ZnO, but a clear trend could not be found through the application of both foliar fertilizers (Table 2). In the leaves of Moscatel, a clear trend also could not be detected for Cu and K (Table 2), but the lowest contents were found after foliar spraying with ZnO or ZnSO_4_ (450 g ha^−1^ and 150 g ha^−1^, respectively). In the grapes of Castelão, the concentrations of K did not vary significantly, but in Moscatel the highest value was found in the control and the lowest after application of ZnSO_4_ (150 g ha^−1^).

The contents of Fe in the leaves of Castelão, although showing some variations among treatments, did not vary significantly after application of both foliar fertilizers, and in Moscatel only a significantly higher concentration was found with ZnSO_4_ (150g ha^−1^) (Table 2).

In the leaves of Moscatel, the levels of S and P were significantly lower and the highest values were in the control and ZnO (450 g ha^−1^), respectively (Table 2). Moreover, in the leaves of Castelão the lowest amounts of S and P were found with ZnSO_4_ (150g ha^−1^) and the highest with ZnO (450 g ha^−1^ and 150 g ha^−1^, respectively). Concerning the grapes of Moscatel, the lowest and highest contents of S were found with 900 g ha^−1^ ZnO and 450 g ha^−1^ ZnSO_4_, whereas in Castelão they were found with 900 g ha^−1^ ZnSO_4_ and 150 g ha^−1^ ZnO (Table 2). Concerning P, in Moscatel the lowest and highest values were detected with 150 g ha^−1^ ZnSO_4_ and the control, whereas for Castelão they were detected with the control and 150 g ha^−1^ ZnO (Table 2).

Between the controls of both varieties, the leaves of Castelão showed significantly higher values of Zn, Fe, S and P, but the opposite occurred with Cu and Ca and significant differences could not be found for K (Table 2). Moreover, heterogeneous trends were found among the remaining treatments (Table 2). The lowest levels of Zn between each treatment for both varieties occurred in Castelão with ZnO (150 g ha^−1^ and 900 g ha^−1^) and ZnSO_4_ (450 g ha^−1^ and 900 g ha^−1^), whereas the opposite occurred with the lowest treatment with ZnSO_4,_ and significant differences could not be found with the application of ZnO at 450 g ha^−1^. The amount of Cu remained significantly lower for all treatments of Castelão. The amount of Fe did not vary significantly with ZnO (150 g ha^−1^ and 900 g ha^−1^) and ZnSO_4_ (450 g ha^−1^ and 900 g ha^−1^), but opposite trends were found between both varieties with ZnO (450 g ha^−1^) and ZnSO_4_ (150 g ha^−1^). The contents of Ca significantly prevailed in Moscatel for all treatments (except with foliar spraying of ZnO at 900 g ha^−1^). Relative to the amounts of K among the treatments of each variety, significant differences were found in Castelão and Moscatel (excepting 900 g ha^−1^ ZnO and 450 g ha^−1^ ZnSO_4_). Concerning S, between each treatment of both varieties, significantly higher values were found for Castelão through the application of the different concentrations of both fertilizers (except with ZnSO_4_, after application of 150 g ha^−1^). The levels of P did not vary significantly between each treatment of both varieties after foliar spraying with the different concentrations of fertilizers (except ZnO and ZnSO_4_ with 450 and 900 g ha^−1^, respectively).

Concerning the variations in each treatment of grapes from Castelão and Moscatel, heterogeneous trends were also found after foliar spraying with different concentrations of both fertilizers (Table 2). The control showed significantly higher contents of Zn, Ca and P in Moscatel, and significant differences could not be found for S and K. Regarding Zn, in similar treatments of each variety, higher values were found with ZnO (150 and 900 g ha^−1^) and ZnSO_4_ (150 and 450 g ha^−1^), but in the other treatments significant differences were not detected. Concerning Ca, similar treatments of both varieties did not reveal significant differences (except Castelão that showed lower contents after the ZnSO_4_ application with 900 g ha^−1^). Relative to the amounts of K in the same treatments of each variety, higher values were consistently found in Castelão (except in the control and 150 and 450 g ha^−1^ of ZnSO_4_ and ZnO, respectively). Between both varieties, each similar treatment revealed higher contents of S in Castelão (excepting the control, 450 g ha^−1^ ZnO, as well as 450 and 900 g ha^−1^ ZnSO_4_). Relative to P, between each similar treatment of both varieties, higher contents were found after application of ZnO at 150 g ha^−1^ in Castelão, but the opposite occurred through foliar spraying with ZnSO_4_ at 900 g ha^−1^, and significant differences could not be found for the remaining concentrations of both fertilizers.

### 2.4. Zn Accumulation in Grapes at Harvest

**Figure 3:**
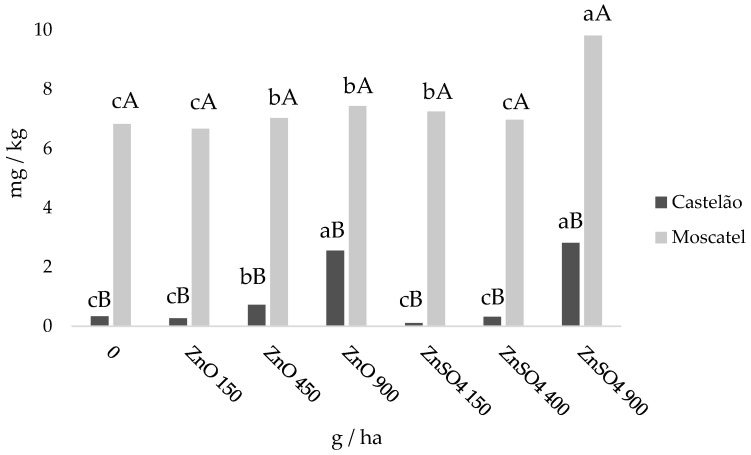
Average ± SE (*n* = 3) of Zn concentrations in grapes of *Vitis vinifera*, varieties of Castelão and Moscatel, at harvest. Letters a, b, c indicate significant differences among treatments of each variety, whereas letters A, B refer to significant differences between both varieties for the same treatment (statistical analysis using the single-factor ANOVA test, *p* < 0.05).

At harvest and relative to the control, Zn accumulation in the whole grapes of Moscatel was consistently higher in all treatments, but in Castelão significantly higher values for Zn contents were only detected in ZnO (450 and 900 g ha^−1^) and ZnSO_4_ after foliar spraying with 900 g ha^−1^ (Figure 3). In this context, depending on the concentrations of the sprayed fertilizers, the indexes of Zn enrichment varied between a 2.14- and 8.38-fold increase in varieties of Castelão, whereas they varied between a 1.02- and 1.44-fold increase in Moscatel.

At harvest, the amount of Zn in the dried skin of Castelão only showed a significant increase with ZnO and ZnSO_4_ spraying at 900 g ha^−1^ (about a 2.71- and 1.5-fold increase relative to the control, respectively) (Figure S1; Table 3). Moreover, in Moscatel a clear trend of Zn accumulation could not be found in the skin (Figure S1; Table 3). In the seeds of Castelão, also treated with ZnO (900 g ha^−1^), a significantly higher value was found, whereas in Moscatel a ca. 1.45- and 2.10-fold increase was found in the two highest concentrations of spray with ZnSO_4_ (450 and 900 g ha^−1^, respectively) (Figure S1; Table 3).

### 2.5. Physicochemical Characteristics and Colorimetric Analysis of Grapes

At harvest, the dry weight of grapes ranged (in %) between about 16 and 23, but did not vary among treatments of each variety or in each treatment between varieties (Table S1). Moreover, although total soluble solids did not vary among treatments in Castelão, significant differences were found for Moscatel (relative to the control, higher values were found for all treatments of ZnO and with 150 and 900 g ha^−1^ of ZnSO_4_) (Table S1). Between varieties, a comparative analysis between the value of each treatment revealed significant differences for all treatments, except the ZnO (at 150 g ha^−1^) and ZnSO_4_ (at 150 and 900 g ha^−1^) treatments having systematically lower values in Moscatel (Table S1).

Concerning the colorimeter parameters (Table S2), L did not vary among treatments or between each treatment of both varieties (except ZnO at 450 g ha^−1^ and ZnSO_4_ at 450 and 900 g ha^−1^). Parameters a * and b * did not vary among treatments in each variety (except a * with ZnSO_4_ at 450 g ha^−1^ in Moscatel). Relative to Castelão, in the grapes of each treatment of Moscatel, a * revealed significantly lower values (except ZnO at 150 g ha^−1^), but the opposite occurred in all treatments with b* (Table S2).

### 2.6. Zn Accumulation in Wine

After winemaking, compared to the control, the contents of Zn increased in both varieties and in all treatments (ZnO and ZnSO_4_ at 450 and 900 g ha^−1^), although non-significantly in Castelão (Table 4). The wine of Castelão showed a 1.34-fold increase, whereas in Moscatel a 3.57-fold increase was found (Table 4). The highest Zn enrichment was found through the application of ZnSO_4_ (at 900 g ha^−1^) in Moscatel, but in Castelão the highest increase was detected (although non-significantly) with ZnO at 900 g ha^−1^ (Table 4).

## 3. Discussion

Mineral and organic components of soils, as well as their chemical and biological processes, interfere with plant productivity, but the bioavailability of nutrients throughout the entire pathway, from soils to plants, also depends on the relief and type of land use. In fact, the slope and slope length of soils are important factors that control the intensity and frequency of surface runoff and, therefore, sediment/fertilizer losses. In this context, the slope of both vineyards (i.e., Lagameças and Lau Novo), having (Table 1) similar areas of low and moderate surface drainages (i.e., classes 1 and 2, thus with ranging slopes between 0–20%) on a percentual basis, determined low soil erosion, as well as a lower rate of alluviums and higher retention of organic matter (which additionally favors water retention) [46]. Nevertheless, although Zn is very mobile in most soils, the higher pH and electric conductivity of Lagameças coupled to significantly higher levels of Ca and Fe can induce higher adsorption and precipitation of Zn by iron oxide coated carbonates (thus holding Zn quite strongly in the soil), compared to Lau Novo [33,47,48]. Besides, the significantly higher level of organic matter of Lagameças (Table 1), which implicates the accumulation of predominant amounts of colloid materials (e.g., organic compounds as amino acids, hydroxy acids), is efficient in complexing Zn and lowering its solubility in alkaline soils (thus, leading to the precipitation of Zn in the form of Zn (OH)_2_ or ZnCO_3_), as well as for Mn, Fe and Ca [49,50]. Comparatively to Lau Novo, these conditioned parameters in the soil of Lagameças implied a higher energy expenditure for root uptake of the Castelão vine (as seen by its significantly higher electrical conductivity, even though the pH is suitable for viticulture in both fields). Nevertheless, although the vineyard of Lau Novo was more suitable for grape production, the physical and chemical composition of irrigation water could be a limitation due to its effects on the soil (waterproofing and/or alkalization) and on the promotion of toxicity to the viticulture. However, the irrigation water of Lau Novo further kept the more favorable conditions for viticulture, as it did not represent a danger of alkalinizing the soil (because it has a low sodium concentration) and can be used in medium-degree leaching conditions and in vines, as they have moderate tolerance to salts. This pattern is further reinforced as this water is sub-saturated with calcium carbonate and has a pH of 6.2, a pHe of 9.3 and an ISL (Langelier saturation index) of −0.31.

After three leaf sprayings with ZnSO_4_ and ZnO, comparatively to the control, both varieties revealed an absence of significant variations of Pn and gs, indicating that at this point the mobilization of photoassimilates was not affected (Figure 2). In fact, non-toxic Zn enrichment of plant species can even lead to increasing rates of net photosynthesis (Pn), transpiration (E) and stomatal conductance (gs) due to Zn’s role in chlorophyll formation and carbonic anhydrase activity (as this enzyme facilitates the diffusion of CO_2_ into chloroplasts) [51]. Furthermore, Zn is involved in stomatal opening since carbonic anhydrase is necessary for maintaining adequate HCO_3_ and K^+^ uptake by the guard cells [51]. Nevertheless, at the end of the productive cycle, Pn and gs decreased in both varieties after leaf spraying with all doses of ZnSO_4_ and ZnO (Figure 2), which suggest that the threshold of Zn toxicity was nearby. Indeed, slightly toxic levels of Zn can decrease Pn and E due to gs reduction without visible symptoms, but cause limitations in other physiological and chemical processes (namely, the physical structure of mesophyll cells and stomata, activity of the carbonic anhydrase enzyme and implications in Mg^2+^ uptake) [52]. Besides, there is evidence that a Zn excess increases pectin and callose content, binding this nutrient excess in the cell wall and immobilizing it (thus, warranting that Zn does not enter into the cytoplasm, which can also cause a growth inhibition) [53]. Moreover, decreased Pn, E and gs contributed to the decrease of iWUE (Figure 2). However, the effect of other factors (namely, hot temperatures and low relative humidity) that can also inhibit photosynthesis through the stomatal closure and non-stomatal inhibitions (e.g., biochemical reactions) cannot be excluded [54], since at the end of the productive cycle both vineyards overlapped with hot summer days [55]. Indeed, stomatal reduction occurs under severe temperature [56], which is often a gradual process that avoids water loss over transpiration, and affects the diffusion of CO_2_ to the carboxylation sites in the chloroplast [57].

Zinc fertilization, which has high phloem mobility in vine [58], is well-known to increase this nutrient accumulation in plant organs [59,60,61,62]. However, at the beginning of fruit development (e.g., after the second spraying), the increased accumulation of Zn in the leaves of Castelão and Moscatel sprayed with both fertilizers was found to be more effective than this nutrient uptake from the soils and triggered the highest kinetics of Zn accumulation in leaves and grapes independently of the edaphic conditions of both vineyards (Table 2). Besides, although disagreement exists over whether Zn mobilization is an active or passive process [33,63,64,65], as previously found in other grapevines [58,59,60,61] at this development stage, ZnO was the most effective in both varieties (Table 2), suggesting a metabolic control in leaves and grapes. In addition, instead of what was seen in previous reports [33,66,67], the imbalance of Zn accumulation did not limit Fe and Cu contents in the leaves of both varieties (Table 2), suggesting different carrier transport metabolisms (namely, interference in chelation processes until accumulation). Moreover, whereas an antagonistic accumulation prevails between Ca and Zn in several plant species, namely dry bean [68], the opposite trend found in the leaves of both genotypes suggests a common mobilization pathway that does not prevail in grapes (Table 2). Similarly, as found by [69] working with corn, in both varieties after the second foliar spraying, K accumulation in leaves positively correlated with the increasing accumulation of Zn (except with ZnO at 900g ha^−1^), clearly further pointing to a synergistic accumulation behavior that also does not prevail in grapes (Table 2). The absence of an antagonism for Zn and S accumulation in leaves and grapes of both varieties (Table 2), as found in Chinese cabbage [70] and for P [67,71,72,73,74], suggest a high metabolic specificity for vines, involving uptake, translocation and mobilization of Zn.

Although grapes are highly sensitive to Zn deficiency [75], despite the significantly different contents of this nutrient between the control of both varieties at harvest (Figure 3), the rates of photoassimilates only slightly varied at the end of the productive cycle of both varieties (Figure 2) and visible symptoms of deficiency [75,76] in the grapevines (namely, chlorosis, necrotic spots, the contraction of plants and little leaf) did not occur. Moreover, relative to Moscatel and independent of the fertilizers applied, all treatments of Castelão revealed significantly lower levels of Zn (Figure 3). Accordingly, independent of genotype specificity, data suggests that the higher pH, electric conductivity and levels of Ca, Fe and organic matter (Table 1) decreased the exchangeable rate of Zn through the promotion of tightly bound fractions of this nutrient in the soil [77] of Lagameças, therefore limiting the uptake rates from roots. In this context, a combined uptake of Zn from soils, at different rates in both varieties, and foliar spraying determined nutrient movement/absorption across the cuticle (e.g., a dissolution-diffusion process) and/or through the stomatal cavity [78,79], increasing the efficiency of Zn accumulation in grapes at harvest (Figure 3). Still, relative to Moscatel, the higher accumulation of Zn in the skin of Castelão in the highest treatments (Table 3; Figure S1), suggests a lower rate of Zn binding to light organic compounds linked to its mobility in the pulp [80,81] and a higher deposition in the seeds. Nevertheless, the highest concentrations of ZnO and ZnSO_4_ revealed the overall efficacy of Zn movement linked to its subsequent loading into the foliar vascular systems and translocation via the phloem of primary veins to other plant tissues [79,82,83,84], determining the highest concentrations in the grapes of both varieties (Figure 3). Considering identical concentrations of each foliar fertilizer, a comparative analysis of Zn accumulation in grapes of each variety revealed similar efficiencies (Table 3) for stomata and/or cuticle movement to the apoplast over time [85]. However, although both chemical forms are the primary forms of Zn fertilizer used with plants [86,87], ZnSO_4_ has a high solubility and high rate of absorption (which can induce phytotoxicity) [88], whereas ZnO reveals (contrarily to grapes) more of a greater positive impact and accumulation in several other plant species than ZnSO_4_ [89,90,91,92].

At harvest, the absence of significant variations in biomass among the different Zn-treated grapes and between both varieties (Table S1) further indicates that the threshold of toxicity was not reached. However, although in different production regions grapes for wine production are harvested according to different criteria (namely, depending on the type of wine), they also derive from the respective glucometric degree (Table S1). In fact, wine production involves the transformation of grape sugar into alcohol and secondary products (namely, organic acids, polyphenolic compounds, anthocyanins and volatile compounds) [93,94,95]. In this context, although the action of the different Zn treatments on the glucometric degree remained residual in Castelão, ZnO and ZnSO_4_, in general, accentuated the total soluble solid content in Moscatel, which is a favorable aspect for wine production (Table S1). Nevertheless, as reported in different species of *Vitis*, the range for total soluble sugar varies between 13.7 and 31.5 °Brix [96], which effectively occurred in all treatments of both varieties (Table S1). Besides, the color change in grapes is accompanied by physical changes as they mature (e.g., berries become turgid, acquiring some elasticity and softening due to the loss of rigidity of the skin and pulp cell walls), increasing the content of the two main sugars, which are glucose and fructose [96]. Still, among all the Zn treatments, the colorimetric parameters of Castelão and Moscatel showed values similar to those mentioned in [97] that, in 78 varieties of grapes, found that L * ranged from 17.74 to 60.27, a * values ranged from −17.19 to 18.11 and b * values ranged from −0.77 to 31.84. Besides, relative to the control, the colorimetric parameters did not vary significantly among treatments of each variety (Table S2), which further indicate the overall quality of the Zn-treated grapes with both fertilizers. Indeed, color is also a good indicator since grapes with low pigment contents are also deficient in sugar and excessively acidic. In this context, the accumulation of Zn in the wine of both varieties (Table 4) followed the content of this nutrient in grapes (Figure 3), prevailing in Moscatel submitted to ZnSO_4_ without surpassing the threshold of toxicity appointed for human consumption (5 mg·L^−1^) [98]. The enrichment of these food products, upon human consumption, can reinforce the biochemical and physiological functions linked with Zn, contributing to better health [99].

## 4. Materials and Methods

### 4.1. Experimental Fields

*Vitis vinifera* varieties, Castelão and Moscatel, were produced in the vineyards of Lagameças and Lau Novo, located in Setubal, Portugal (GPS coordinates 38°36′01.19″ N; 8°48′18.18″ W and 38°35′47.113″ N; 8°40′46.651″ W, respectively).

After flowering (on 16 June), three leaf spraying applications with ZnO or ZnSO_4_ (150, 450 and 900 g ha^−1^) were performed with 14–21 day intervals. Control vines were sprayed with water. Harvest was performed by 24 and 25 September for Castelão and Moscatel, respectively. Between 16 June and 25 September, maximum and minimum mean temperatures ranged between 28 and 16.6 °C.

### 4.2. Orthophotomap

On 1 August 2018, for each plot (i.e., prior to foliar spraying) of both vineyards, data was collected using a drone (DJI Phantom Pro V2.0), with high-definition and multi-sector RGB (i.e., with three electromagnetic spectra bands—red, green and blue) and Parrot Sequoia (i.e., with five electromagnetic spectra bands—NIR, REG, green, red and RGB) cameras that were used to produce orthophotomaps. Calibration of the multispectral Parrot Sequoia camera further considered the environmental brightness conditions. Images were processed in a workstation (AORUS, GIGA-BYTE Technology Co., Ltd.—2019), to produce the final mapping. To assess the general morphology and surface water drainage areas of the experimental fields, Agisoft PhotoScan Professional (version 1.2.6, software from 2016, ESRI from 2011 and ArcGIS Desktop—Release 10 from Redlands, CA: Environmental Systems Research Institute) was used. The evaluation of the drainage areas of surface water was carried out according to [100]. The highest class corresponded to the land that, due to its morphology, enhanced the surface runoff of water and did not promote infiltration. Conversely, the lower class corresponded to flattened surfaces as potential infiltration areas, since they promote the accumulation of surface water.

### 4.3. Soil and Irrigation Water Analysis

The content of organic matter in the soils of the vineyards was determined in 28 samples (about 100 g were collect from the surface to a 30 cm depth). Samples were sieved (2.0 mm mesh to remove stones, coarse materials and other debris) and the weight recorded after drying (at 105 °C for 24 h, followed by a 1 h desiccation) for quantification of the dry mass and percentage of moisture.

To determine the content of organic matter, samples were heated to 550 °C for 4 h (i.e., until a constant weight) and, after removal from the muffle (at 100 °C), desiccated until room temperature was reached (approximately 1 h). Samples were then weighed to determine the percentage of organic matter.

Using a potentiometer, pH and electrical conductivity of soil samples were determined. After mixing at a ratio of 1:2.5 (g _soil_ mL^−1^ _water milli-q_) and stirring for 1 h (at 25 °C for 30 min) in a thermal bath, determinations were carried out after decantation of the supernatant [101].

Mineral elements of soil samples were quantified using an XRF analyzer (model XL3t 950 He GOLDD+) under a helium atmosphere (Niton Thermal Scientific, Munich, Germany), according to [102].

Water quality was analyzed considering physical (pH, temperature and electrical conductivity) and chemical (bicarbonate, sulfate, chloride, sodium, calcium, magnesium, potassium, nitrate and phosphate) parameters. Electrical conductivity (EC) and pH were determined using a Consort Multiparameter analyzer (C 6030) and SP21 (pH) and SK20T (CE) electrodes. Calcium, Na, K and Mg ions were quantified using a Metrohm (Model 761 Compact IC) chromatograph, equipped with a column and pre-column (Metrosep cation 1-2, 6.1010.000) using an eluent mixture (4 mM tartaric acid/1 mM dipicolinic acid) at a flow rate of 1.00 mL/min and a sample injection of 10.0 μL. Alkalinity/bicarbonate was determined by titration in 100 mL of water samples, using 0.1 N hydrochloric acid as the titrant in the presence of 0.1% methyl orange [103].

Chloride, sulphate, nitrate and phosphate ions were quantified by photometry (Spectroquant NOVA 60, Merck, Darmstadt, Germany) using specific kits (1.14897, 1.14779, 1.14773 and 1.14842). Water classification considered dominant ions and followed [104]. A sodium adsorption index was determined and related to the electrical conductivity in classes C and S. The Langelier saturation index was also estimated from the pHe (equilibrium pH), at a reference temperature of 20 °C, to determine the fouling or aggressiveness of the water relative to calcium carbonate.

### 4.4. Leaf Gas Exchange Measurements

Leaf gas exchange parameters were determined using 4–6 randomized leaves per treatment on 1 August (1st assessment) and 13 September (2nd assessment), following [105]. Leaf rates of net photosynthesis (Pn), stomatal conductance to water vapor (gs) and transpiration (E) were obtained under photosynthetic steady-state conditions after ca. 2 h of illumination (in the middle morning). A portable open-system infrared gas analyzer (Li-Cor 6400, Li-Cor, Lincoln, NE, USA) was used under environmental conditions, with external CO_2_ (ca. 400 ppm) and PPFD ranging between 1200–1400 µmol m^−2^ s^−1^. Leaf instantaneous water use efficiency (iWUE) was calculated as the Pn-to-E ratio, representing the units of assimilated CO_2_ per unit of water lost through transpiration.

### 4.5. Analysis of Nutrient Contents in Grapes and Leaves

After the 2nd foliar application, nutrient contents were quantified in randomized leaves and grapes (dried at 60 °C, until a constant weight, then ground and processed into pellet) according to [102], using an XRF analyzer (model XL3t 950 He GOLDD+) under He atmosphere (Niton Thermal Scientific, Munich, Germany) [106].

### 4.6. Analysis of Total Zinc Content in Grapes and Wine

After harvest, randomized grapes were washed, dried at 60 °C until a constant weight and ground in an agate mortar. Thereafter, an acid digestion procedure was performed with a mixture of HNO_3_^−^:HClO_4_ (4:1), according to [107], followed by filtration. Zinc content was measured in grapes and wine with an atomic absorption spectrophotometer model, the Perkin Elmer AAnalyst 200 (Waltham, Massachusetts, MA, USA), fitted with a deuterium background corrector and using the AA WinLab software program.

### 4.7. Analysis of Zinc Content in Grape Tissues

The location of Zn in the skin and seeds of grapes, collected at harvest, was determined with the µ-EDXRF system (M4 Tornado™, Bruker, Germany), according to [108]. The X-ray generator was operated at 50 kV and 100 µA without the use of filters, to enhance the ionization of low-Z elements. For a better quantification of Zn, a set of filters between the X-ray tube and the sample, composed of three foils of Al/Ti/Cu (with a thickness of 100/50/25 µm, respectively) was used. All the measurements with filters were performed with a 600 µA current. Detection of fluorescence radiation was performed by an energy-dispersive silicon drift detector, XFlash™, with a 30 mm^2^ sensitive area and energy resolution of 142 eV for Mn Kα. Measurements were carried out under 20 mbar vacuum conditions. These point spectra were acquired for a duration of 200 s.

### 4.8. Morphometric and Colorimetric Analyses

Dry weight was measured by considering three randomized grapes per treatment.

Total soluble solids were measured in the grapes’ juice, using a digital refractometer from Atago (Atago, Tokyo, Japan); the obtained values were expressed as °Brix.

Colorimetric parameters, using a fixed wavelength, adopted the methodology described by [57]. Brightness (L *) and chromaticity parameters (a * and b * coordinates) were obtained with a Minolta CR 300 colorimeter (Minolta Corp., Ramsey, NJ, USA) coupled to a sample vessel (CR-A504). Using the illuminant D_65_, the system of the Commission Internationale d’Éclaire (CIE) was applied. The parameter L * represented the brightness of the sample, translating the variation of the tonality between dark and light, with a range between 0 (black) and 100 (white). Parameters a * and b * indicated color variations between red (+60) and green (−60), and between yellow (+60) and blue (−60), respectively. The approximation of these coordinates to the null value are consider neutral colors such as white, gray and black. Measurements were carried out at harvest, considered in triplicates of three independent series.

### 4.9. Winemaking

After destemming (50 kg) and pressing the grapes, sulfur dioxide was added to the must (18 mL) and, after 24 h of rest at 6 °C, Springarom (18 g) was added to the vat. The yeast was hydrated with water at 37 °C (1:10), and after 20 min it was added to the wort in the vat, followed by homogenization of the mixture. The temperature and density of the mixture were then regularly checked and PVPP/Polyvinylpolypyrrolidone—Divergan F (12 g) was applied when the density reached 1060 g/cm^3^; DAP—Diammonium phosphate (12 g) was applied at the peak of fermentation (density between 1030–1040 g/cm^3^) and when the density reached 1000 g/cm^3^; sulfur dioxide (3 mL) was applied when the density reached 990 g/cm^3^. The wine was then filtered, followed by bottling.

### 4.10. Statistical Analysis

Data were statistically analyzed using a one-way or two-way ANOVA to assess differences between treatments and experimental periods, followed by a Tukey’s test for mean comparison. A 95% confidence level was adopted for all tests.

## 5. Conclusions

The threshold of toxicity was not reached through foliar application of ZnO or ZnSO_4_ at concentrations of 900 g ha^−1^ on Castelão and Moscatel grapes, but although the synthesis of photoassimilates was not affected in the mid-term of the grapes’ development, by the end of the productive cycle inhibitory effects on Pn and gs limited the water use efficiency. Independently of Zn fertilization through foliar spraying, the higher pH, electric conductivity and level of organic matter in the soils of the vineyard of Castelão, coupled with significantly higher levels of Ca and Fe, determined higher energy expenditure for root uptake of Castelão, determining (eventually in conjunction with genotype characteristics) significantly lower levels of Zn accumulation in grapes. Moreover, during fruit development, the increased accumulation of Zn in the leaves of Castelão and Moscatel sprayed with both fertilizers was found to be more effective (especially with ZnO) and did not limit Fe and Cu contents, further inducing a synergistic accumulation between Ca and Zn (thus, suggesting a common mobilization pathway that did not prevail in grapes). In addition, a combined uptake of Zn from soils, at different rates in both varieties, and foliar spraying determined nutrient movement/absorption across the cuticle and/or through the stomatal cavity, increasing the efficiency of Zn accumulation in grapes at harvest. Still, the higher accumulation of Zn in the skin of Castelão in the highest treatments pointed to a lower rate of Zn binding to light organic compounds that are linked to its mobility in the pulp and a higher deposition in the seeds. To a different extent, the glucometric degree of grapes can become accentuated by both Zn fertilizers, which favors winemaking. Besides, the accumulation of Zn in wine from both varieties, as follows the contents of this nutrient in grapes (although prevailing in Moscatel submitted to ZnSO_4_), allowed the development of a new functional food product.

## Figures and Tables

**Table 1:** Characterization of soils and irrigation water in the vineyards of Lagameças and Lau Novo fields. Letters a, b indicate significant differences of each parameter between both vineyards (statistical analysis using the single-factor ANOVA test, *p* ≤ 0.05).

Field	Ability to Accumulate or Drain Surface Water										
	Slope Class (%)		Surface Drainage			Area (m^2^)		Area (%)			
Lagameças	1—(0–5%)		Low			437.7		49.38			
	2—(5–20%)		Moderate			448.4		50.59			
	3—>20%		High			0.2		0.02			
Lau Novo	1—(0–5%)		Low			589.9		34.87			
	2—(5–20%)		Moderate			1080.5		63.86			
	3—>20%		High			21.4		1.27			
	Soil analysis (0–30 cm deep) (*n* = 28)										
	pH	Electrical Conductivity	Organic Matter	Ca	K	Mg	P	Fe	S	Zn	Mn
		μS cm^−1^	%						mg/kg		
Lagameças	7.08 ± 0.08 a	100.83 ± 7.11 a	1.48 ± 0.10 a	0.28 ± 0.03 a	2.53 ± 0.05 b	0.07 ± 0.04 a	0.14 ± 0.00 b	0.47 ± 0.03 a	36.82 ± 2.28 a	34.65 ± 3.42 a	191.41 ± 13.90 a
Lau Novo	72.05 ± 2.90 b	6.80 ± 0.06 b	1.09 ± 0.04 b	0.17 ± 0.01 b	3.20 ± 0.05 a	0.07 ± 0.00 a	0.20 ± 0.02 a	0.26 ± 0.01 b	25.03 ± 8.12 a	23.77 ± 1.88 b	145.11 ± 6.98 b
	Water analysis										
	pH	Electrical Conductivity	Ca^2+^	K^+^	Mg^2+^	Na^+^	Cl^−^	HCO_3_^−^	SO_4_^2−^	NO_3_^−^	PO_4_^3−^
		μS cm^−1^	mg L^−1^ (meq L^−1^)								
Lau Novo	6.27	252.01	8.51 (0.40)	3.98 (0.12)	4.39 (0.31)	21.73 (0.93)	34.70 (0.90)	34.77 (0.51)	33.10 (0.63)	17.40 (0.21)	<1.5 (<0.04)

**Table 2:** Average ± SE (*n* = 3) of nutrient concentration in leaves and grapes of *Vitis vinifera,* varieties Castelão and Moscatel, after the second foliar application. Letters a, b, c, d, e indicate significant differences within the same column and variety, whereas letters A, B refer to significant differences between both varieties for the same treatment (statistical analysis using the single-factor ANOVA test, *p* ≤ 0.05).

				Leaves				
	Treatments	Zn	Cu	Fe	Ca	K	S	P
		mg/kg	mg/kg	mg/kg	%	%	%	%
Castelão	Control	33.87 ± 1.35 eA	61.91 ± 4.51 bB	110.47 ± 9.38 aA	2.78 ± 0.06 dB	2.60 ± 0.05 c,dA	0.65 ± 0.02 aA	0.25 ± 0.01 bA
	ZnO (150 g ha^−1^)	98.89 ± 2.03 dB	81.20 ± 0.80 aB	74.97 ± 0.90 aA	3.60 ± 0.09 aB	2.95 ± 0.07 bA	0.61 ± 0.01 a,bA	0.31 ± 0.00 aA
	ZnO (450 g ha^−1^)	309.44 ± 1.49 bA	72.08 ± 3.01 a,bB	111.62 ± 5.94 aA	3.21 ± 0.10 b,cB	3.68 ± 0.06 aA	0.65 ± 0.02 aA	0.31 ± 0.01 aB
	ZnO (900 g ha^−1^)	490.55 ± 12.32 aB	40.27 ± 0.72 cB	100.54 ± 12.19 aA	3.71 ± 0.06 aA	2.39 ± 0.06 dA	0.54 ± 0.03 b,cA	0.31 ± 0.02 aA
	ZnSO_4_ (150 g ha^−1^)	284.22 ± 8.59 bA	61.99 ± 5.26 bB	103.69 ± 12.73 aB	3.12 ± 0.03 cB	3.01 ± 0.04 bA	0.52 ± 0.01 cA	0.24 ± 0.01 bA
	ZnSO_4_ (450 g ha^−1^)	113.26 ± 1.49 dB	66.47 ± 2.19 a,bB	115.80 ± 6.36 aA	3.51 ± 0.03 a,bB	2.47 ± 0.01 c,dA	0.58 ± 0.01 a,b,cA	0.29 ± 0.01 a,bA
	ZnSO_4_ (900 g ha^−1^)	196.35 ± 3.40 cB	58.72 ± 2.78 bB	96.85 ± 5.69 aA	3.09 ± 0.02 cB	2.68 ± 0.05 cA	0.55 ± 0.01 b,cA	0.26 ± 0.00 bB
Moscatel	Control	17.47 ± 3.09 eB	1883.46 ± 4.81 b,cA	60.29 ± 4.47 bB	3.22 ± 0.06 eA	2.35 ± 0.05 a,bA	0.42 ± 0.00 cB	0.20 ± 0.00 dB
	ZnO (150 g ha^−1^)	135.64 ± 3.72 cA	2121.86 ± 67.14 a,bA	74.42 ± 4.77 bA	4.19 ± 0.11 b,cA	2.48 ± 0.04 a,bB	0.50 ± 0.02 bB	0.31 ± 0.01 b,cA
	ZnO (450 g ha^−1^)	289.95 ± 11.98 bA	1492.61 ± 45.03 cA	67.58 ± 9.79 bB	4.53 ± 0.07 a,bA	2.65 ± 0.06 aB	0.57 ± 0.01 aB	0.37 ± 0.01 aA
	ZnO (900 g ha^−1^)	584.25 ± 6.39 aA	2217.30 ± 20.80 a,bA	93.73 ± 10.83 bA	3.63 ± 0.03 d,eA	2.30 ± 0.06 bA	0.43 ± 0.00 b,cB	0.37 ± 0.01 a,bA
	ZnSO_4_ (150 g ha^−1^)	73.22 ± 3.83 dB	2264.28 ± 158.37 a,bA	168.73 ± 2.91 aA	3.89 ± 0.05 c,dA	2.27 ± 0.01 bB	0.46 ± 0.03 b,cA	0.26 ± 0.02 cA
	ZnSO_4_ (450 g ha^−1^)	183.94 ± 5.87 cA	2445.83 ± 67.57 aA	96.46 ± 3.36 bA	3.98 ± 0.10 c,dA	2.41 ± 0.09 a,bA	0.49 ± 0.01 b,cB	0.31 ± 0.02 cA
	ZnSO_4_ (900 g ha^−1^)	323.64 ± 22.52 bA	1593.96 ± 144.08 cA	98.19 ± 15.91 bA	4.70 ± 0.15 aA	2.30 ± 0.11 bB	0.48 ± 0.02 b,cB	0.29 ± 0.01 cA
				Grapes				
	Treatments	Zn	Cu	Fe	Ca	K	S	P
		mg/kg	mg/kg	mg/kg	%	%	%	%
Castelão	Control	7.19 ± 0.73 cB	n.d.		0.53 ± 0.01 b,cB	2.02 ± 0.07 aA	0.16 ± 0.00 b,cA	0.18 ± 0.01 dB
	ZnO (150 g ha^−1^)	9.29 ± 0.36 b,cB			0.81 ± 0.06 aA	2.64 ± 0.13 aA	0.21 ± 0.01 aA	0.28 ± 0.01 aA
	ZnO (450 g ha^−1^)	9.99 ± 1.59 b,cA			0.77 ± 0.06 a,bA	2.36 ± 0.26 aA	0.17 ± 0.01 b,cA	0.21 ± 0.00 c,dA
	ZnO (900 g ha^−1^)	16.03 ± 0.34 aB			0.81 ± 0.05 aA	2.18 ± 0.01 aA	0.20 ± 0.01 a,bA	0.26 ± 0.01 a,bA
	ZnSO_4_ (150 g ha^−1^)	8.45 ± 0.75 b,cB			0.63 ± 0.07 a,b,cA	2.20 ± 0.27 aA	0.20 ± 0.01 a,b,cA	0.23 ± 0.01 b,cA
	ZnSO_4_ (450 g ha^−1^)	7.84 ± 0.41 b,cB			0.66 ± 0.05 a,b,cA	2.05 ± 0.04 aA	0.19 ± 0.00 a,b,cA	0.23 ± 0.01 b,cA
	ZnSO_4_ (900 g ha^−1^)	11.44 ± 1.08 bA			0.48 ± 0.03 cB	2.18 ± 0.02 aA	0.16 ± 0.01 cA	0.20 ± 0.00 c,dB
Moscatel	Control	12.58 ± 0.49 cA	n.d.		0.77 ± 0.05 a,bA	2.06 ± 0.03 aA	0.16 ± 0.00 a,bA	0.29 ± 0.00 aA
	ZnO (150 g ha^−1^)	11.38 ± 0.56 cA			0.73 ± 0.05 a,bA	1.83 ± 0.05 a,bB	0.16 ± 0.00 a,bB	0.23 ± 0.00 b,cB
	ZnO (450 g ha^−1^)	13.42 ± 0.09 b,cA			0.64 ± 0.02 bA	1.88 ± 0.07 a,bA	0.16 ± 0.00 a,bA	0.20 ± 0.01 c,dA
	ZnO (900 g ha^−1^)	21.16 ± 1.74 aA			0.89 ± 0.03 aA	1.87 ± 0.09 a,bB	0.15 ± 0.01 bB	0.22 ± 0.01 b,c,dA
	ZnSO_4_ (150 g ha^−1^)	11.47 ± 0.13 cA			0.81 ± 0.06 a,bA	1.56 ± 0.10 bA	0.15 ± 0.01 a,bB	0.19 ± 0.01 dA
	ZnSO_4_ (450 g ha^−1^)	16.73 ± 0.86 bA			0.75 ± 0.04 a,bA	1.83 ± 0.06 a,bB	0.18 ± 0.01 aA	0.25 ± 0.01 a,bA
	ZnSO_4_ (900 g ha^−1^)	14.41 ± 0.31 b,cA			0.65 ± 0.03 bA	1.58 ± 0.07 bB	0.16 ± 0.00 a,bA	0.23 ± 0.01 b,c,dA

n.d. = not detected.

**Table 3:** Average ± SE (*n* = 3) of Zn concentrations of skin and seeds of *Vitis vinifera* varieties Castelão and Moscatel. Letters a, b, c, d indicate significant differences within the same column and variety, whereas letters A, B refer to significant differences among treatments for the same variety (a, b, c, d) and between varieties for the same treatment (A,B) (statistical analysis using the single-factor ANOVA test, *p* ≤ 0.05).

Treatments	Zn (mg/kg)			
	Skin		Seeds	
	Castelão	Moscatel	Castelão	Moscatel
Control	20.06 ± 1.00 cB	38.34 ± 1.92 aA	16.02 ± 0.80 cA	13.57 ± 0.68 cB
ZnO (150 g ha^−1^)	16.61 ± 0.83 cB	31.12 ± 1.56 bA	10.96 ± 0.55 dB	15.64 ± 0.78 cA
ZnO (450 g ha^−1^)	21.78 ± 1.09 cB	27.49 ± 1.37 bcA	20.87 ± 1.04 bA	14.59 ± 0.73 cB
ZnO (900 g ha^−1^)	54.37 ± 2.72 aA	22.68 ± 1.13 cB	27.79 ± 1.39 aA	16.82 ± 0.84 bcB
ZnSO_4_ (150 g ha^−1^)	21.48 ± 1.07 cA	21.17 ± 2.71 cA	10.63 ± 0.53 dA	9.82 ± 0.49 dA
ZnSO_4_ (450 g ha^−1^)	17.53 ± 0.88 cB	32.16 ± 1.61 abA	15.48 ± 0.77 cB	19.70 ± 0.99 bA
ZnSO_4_ (900 g ha^−1^)	30.16 ± 1.51 bA	23.25 ± 1.16 cB	15.73 ± 0.79 cB	28.59 ± 1.43 aA

**Table 4:** Average ± SE (*n* = 3) of zinc concentrations in wine of *Vitis vinifera* varieties Castelão and Moscatel. Letters a, b, c indicate significant differences among treatments in each variety, whereas letters A and B indicate the significant differences between each treatment of both varieties (statistical analysis using the single-factor ANOVA test, *p* ≤ 0.05).

Treatments	Wine	
	Zn (µg L^−1^)	
	Castelão	Moscatel
Control	0.68 ± 0.27 aA	0.54 ± 0.26 cA
ZnO (450 g ha^−1^)	0.77 ± 0.10 aB	1.20 ± 0.08 bA
ZnO (900 g ha^−1^)	0.91 ± 0.08 aA	1.05 ± 0.02 b,cA
ZnSO_4_ (450 g ha^−1^)	0.89 ± 0.02 aB	1.17 ± 0.06 bA
ZnSO_4_ (900 g ha^−1^)	0.82 ± 0.14 aB	1.92 ± 0.10 aA

## Data Availability

Not applicable.

## Supplementary Materials

The following supporting information can be downloaded at: https://www.mdpi.com/article/10.3390/plants11111399/s1, Figure S1: Identification of skin (A) and seed (B) regions in grapes of *Vitis vinifera* varieties Castelão and Moscatel, where Zn content was measured with the µ-EDXRF system (M4 Tornado™, Bruker, Germany); Table S1: Average ± SE (*n* = 3) of dry weight and total soluble solids (expressed as °Brix) in grapes of *Vitis vinifera* varieties Castelão and Moscatel. Letters a, b indicate significant differences among treatments in each variety, whereas letters A and B indicate the significant differences between varieties in each treatment (statistical analysis using the single-factor ANOVA test, *p* < 0.05); Table S2: Average ± SE (*n* = 3) of colorimeter parameters of the skin of grapes of *Vitis vinifera* varieties Castelão and Moscatel. Letters a, b indicate significant differences among treatments in each variety, whereas letters A and B indicate the significant differences between each parameter for both varieties in each treatment (statistical analysis using the single-factor ANOVA test, *p* < 0.05).

## References

[1] Barak P., Helmke P.A., Robson A.D. (1993). The chemistry of zinc. Zinc in Sil and Plants.

[2] Auld D.S. (2001). Zinc coordination sphere in biochemical zinc sites. Biometals.

[3] Klug A. (1999). Zinc finger peptides for the regulation of gene expression. J. Mol. Biol..

[4] Englbrecht C.C., Schoof H., Böhm S. (2004). Conservation, diversification and expansion of C_2_H_2_ zinc finger proteins in the *Arabidopsis thaliana* genome. BMC Genom..

[5] Liu D., Liu Y., Zhang W., Chen X., Zou C. (2017). Agronomic approach of zinc biofortification can increase zinc bioavailability in wheat flour and thereby reduce zinc deficiency in humans. Nutrients.

[6] Webb E.C. (1992). Enzyme Nomenclature, Recommendations of the Nomenclature Committee of the International Union of Biochemistry and Molecular Biology.

[7] Gammoh N.Z., Rink L. (2017). Zinc in infection and inflammation. Nutrients.

[8] Uwitonze A.M., Ojeh N., Murererehe J., Atfi A., Razzaque M.S. (2020). Zinc adequacy is essential for the maintenance of optimal oral health. Nutrients.

[9] IAEA—International Atomic Energy Agency. https://www.iaea.org/opic/annual-report-2018.

[10] Rugeles-Reyes S.M., Cecílio A.B., Aguilar M.A.L., Silva P.H.S. (2019). Foliar application of zinc in the agronomic biofortification of arugula. Food Sci. Technol..

[11] Chasapis C.T., Ntoupa P.S., Spiliopoulou C.A., Stefanidou M.E. (2020). Recent aspects of the effects of zinc on human health. Arch. Toxicol..

[12] Grüngreiff K., Gottstein T., Reinhold D. (2020). Zinc deficiency-An independent risk factor in the pathogenesis of haemorrhagic stroke?. Nutrients.

[13] Andreini C., Banci L., Bertini I., Rosato A. (2006). Counting the zinc-proteins encoded in the human genome. J. Proteome Res..

[14] Beyersmann D., Haase H. (2001). Functions of zinc in signaling, proliferation and differentiation of mammalian cells. Biometals.

[15] Cakmak I. (2008). Enrichment of cereal grains with zinc: Agronomic or genetic biofortification?. Plant Soil.

[16] Bouis H.E., Welch R.M. (2010). Biofortification—A sustainable agricultural strategy for reducing micronutrient malnutrition in the global south. Crop Sci..

[17] Pal V., Singh G., Dhaliwal S.S. (2019). Agronomic biofortification of chickpea with zinc and iron through application of zinc and urea. Commun. Soil Sci. Plant Anal..

[18] Palmgren M.G., Clemens S., Williams L.E., Krämer U., Borg S., Schjørring J.K., Sanders D. (2008). Zinc biofortification of cereals: Problems and solutions. Trends Plant Sci..

[19] Zulfiqar U., Hussain S., Ishfaq M., Matloob A., Ali N., Ahmad M., Alyemeni M.N., Ahmad P. (2020). Zinc-induced efects on productivity, zinc use eficiency, and grain biofortification of bread wheat under diferent tillage permutations. Agronomy.

[20] Hussain A., Jiang W., Wang X., Shahid S., Saba N., Ahmad M., Dar A., Masood S.U., Imran M., Mustafa A. (2022). Mechanistic impact of zinc deficiency in human development. Front. Nutr..

[21] Erenoglu E.B., Kutman U.B., Ceylan Y., Yildiz B., Cakmak I. (2011). Improved nitrogen nutrition enhances root uptake, root-to-shoot translocation and remobilization of zinc (^65^Zn) in wheat. New Phytol..

[22] Stanton C., Sanders D., Krämer U., Podar D. (2022). Zinc in plants: Integrating homeostasis and biofortification. Mol. Plant.

[23] Moreira A., Moraes L.A.C., dos Reis A.R., Hossain M.A., Kamiya T., Burrit D.J., Tran L.P., Fujiwara T. (2018). The molecular genetics of zinc uptake and utilization efficiency in crop plants. Plant Micronutrient Use Efficiency.

[24] Rose T.J., Impa S.M., Rose M.T., Pariasca-Tanaka J., Mori A., Heuer S., Johnson-Beebout S.E., Wissuwa M. (2013). Enhancing phosphorus and zinc acquisition efficiency in rice: A critical review of root traits and their potential utility in rice breeding. Ann. Bot..

[25] Liu D.Y., Liu Y.M., Zhang W., Chen X.P., Zou C.Q. (2019). Zinc Uptake, Translocation, and remobilization in winter wheat as affected by soil application of Zn fertilizer. Front. Plant Sci..

[26] Xue Y., Yue S., Zhang W., Liu D., Cui Z., Chen X., Ye Y., Zou C. (2014). Zinc, iron, manganese and copper uptake requirement in response to nitrogen supply and the increased grain yield of summer maize. PLoS ONE.

[27] Dwivedi R.S., Randhawa N.S., Bansal R.L. (1975). Phosphorus-zinc interaction: I. Sites of immobilization of zinc in maize at a high level of phosphorus. Plant Soil.

[28] Impa S.M., Morete M.J., Ismail A.M., Schulin R., Johnson-Beebout S.E. (2013). Zn uptake, translocation and grain Zn loading in rice (*Oryza sativa* L.) genotypes selected for Zn deficiency tolerance and high grain Zn. J. Exp. Bot..

[29] Stomph T.J., Jiang W., Van Der Putten P.E.L., Struik P.C. (2014). Zinc allocation and re-allocation in rice. Front. Plant Sci..

[30] Rengel Z., Batten G.D., Crowley D.E. (1999). Agronomic approaches for improving the micronutrient density in edible portions of field crops. Field Crop. Res..

[31] Dhaliwal S.S., Sharma V., Shukla A.K., Verma V., Sandhu P.S., Behera S.K., Singh P., Kaur J., Singh H., Abdel-Hafez S.H. (2021). Interactive effects of foliar application of zinc, iron and nitrogen on productivity and nutritional quality of Indian mustard (*Brassica juncea* L.). Agronomy.

[32] Khoshgoftarmanesh A.H., Schulin R., Chaney R.L., Daneshbakhsh B., Afyuni M. (2010). Micronutrient-efficient genotypes for crop yield and nutritional quality in sustainable agriculture: A review. Agron. Sustain. Dev..

[33] Pendias K., Pendias H. (2001). Trace Elements in Soils and Plants.

[34] Ohnishi M., Furutani R., Sohtome T., Suzuki T., Wada S., Tanaka S., Ifuku K., Ueno D., Miyake C. (2021). Photosynthetic parameters show specific responses to essential mineral deficiencies. Antioxidants.

[35] Broadley M.R., White P.J., Hammond J.P., Zelkp I., Lux A. (2007). Zinc in plants. New Phytol..

[36] Ahmad P., Alymeni M.N., Al-Hugail A.A., Algahtani M.A., Wijaya L., Ashraf M., Kaya C., Bajguz A. (2020). Zinc oxide nanoparticles application alleviates arsenic (As) toxicity in soybean plants by restricting the uptake of as and modulating key biochemical attributes, antioxidant enzymes, ascorbate-glutathione cycle and glyoxalase system. Plants.

[37] Jalal A., Shah S., Filho M.C.M.T., Khan A., Shah T., Ilyas M., Rosa P.A.L. (2020). Agro-Biofortification of zinc and iron in wheat grains. Gesunde Pflanz..

[38] Dutta T., Neelapu N.R.R., Surekha C., Roychoudhury A., Tripathi D.K. (2020). Iron, zinc, and copper application in overcoming environmental stress. Protective Chemical Agents in the Amelioration of Plant Abiotic Stress: Biochemical and Molecular Perspectives.

[39] Kandoliya R.U., Sakarvadiya H.L., Kunjadia B.B. (2018). Effect of zinc and iron application on leaf chlorophyll, carotenoid, grain yield and quality of wheat in calcareous soil of Saurashtra region. Int. J. Chem. Stud..

[40] McKevith B. (2004). Nutritional aspects of cereals. Nutr. Bull..

[41] Ozturk L., Yazici M.A., Yucel C., Torun A., Cekic C., Bagci A., Ozkan H., Braun H.J., Sayers Z., Cakmak I. (2006). Concentration and localization of zinc during seed development and germination in wheat. Plant Physiol..

[42] Aisbitt B., Caswell H., Lunn J. (2008). Cereals–current and emerging nutritional issues. Nutr. Bull..

[43] Li M., Yang X.W., Tian X.H., Wang S.X., Chen Y.L. (2013). Effect of nitrogen fertilizer and foliar zinc application at different growth stages on zinc translocation and utilization efficiency in winter wheat. Cereal Res. Commun..

[44] Abdoli M., Esfandiari E., Mousavi S.B., Sadeghzadeh B. (2014). Effects of foliar application of zinc sulfate at different phenological stages on yield formation and grain zinc content of bread wheat (cv. *Kohdasht*). Azarian J. Agric..

[45] Aisha I., Muhammad Y.A., Mumtaz H., Muhammad A., Rashid A., Ali K. (2015). Effect of micronutrients (zn, cu and b) on photosynthetic and fruit yield attributes of citrus reticulata blanco variety kinnow. Pak. J. Bot..

[46] Teixeira R.F.M., Domingos T., Costa A.P.S.V., Oliveira R., Farropas L., Calouro F., Barradas A.M., Carneiro J.P.B.G. (2011). Soil organic matter dynamics in Portuguese natural and sown rainfed grasslands. Ecol. Model..

[47] Kabata-Pendias A., Mukherjee A.B. (2007). Trace Elements from Soils to Humans.

[48] Racena R., Garcia-Lopez A.M., Delgado A. (2021). Zinc uptake by plants as affected by fertilization with Zn sulfate, phosphorous availability and soil properties. Plants.

[49] Suganya A., Saravanan A., Manivannan N. (2020). Role of Zinc Nutrition for Increasing Zinc Availability, Uptake, Yield, and Quality of Maize (*Zea mays* L.) Grains: An Overview. Commun. Soil Sci. Plant Anal..

[50] Luís I.C., Lidon F.C., Pessoa C.C., Marques A.C., Coelho A.R.F., Simões M., Patanita M., Dôres J., Ramalho J.C., Silva M.M. (2021). Zinc enrichment in two contrasting genotypes of *Triticum aestivum* L. grains: Interactions between edaphic conditions and foliar fertilizers. Plants.

[51] Ahmed N., Ahmad F., Abid M., Ullah M.A. (2009). Impact of zinc fertilization on gas exchange characteristics and water use efficiency of cotton crop under arid environment. Pak. J. Bot..

[52] Saboor A., Ali M.A., Ahmed N., Skalicky M., Danish S., Fahad S., Hassan F., Hassan M.M., Brestic M., Sabagh A.E. (2021). Biofertilizer-based zinc application enhances maize growth, gas exchange attributes, and yield in zinc-deficient soil. Agriculture.

[53] Kaur H., Garg N. (2021). Zinc toxicity in plants: A review. Planta.

[54] Ashraf M., Harris P.J.C. (2013). Photosynthesis under stressful environments: An overview. Photosynthetica.

[55] Moutinho-Pereira J.M., Correia C.M., Gonçalves B.M., Bacelar E.A., Torres-Pereira J.M. (2004). Leaf Gas Exchange and Water Relations of Grapevines Grown in Three Different Conditions. Photosynthetica.

[56] De Oliveira A.C., Pegoraro C., Viana V.E. (2020). The Future of Rice Demand: Quality Beyond Productivity.

[57] Ramalho J.C., Zlatev Z.S., Leitão A.E., Pais I.P., Fortunato A.S., Lidon F.C. (2013). Moderate water stress causes different stomatal and non-stomatal changes in the photosynthetic functioning of *Phaseolus vulgaris* L. genotypes. Plant Biol..

[58] Christensen P., Jensen F. (1976). Foliar uptake of zinc nutritional sprays: A study of application methods, timing, and materials. Report of Research for Fresh Table Grapes.

[59] Christensen P., Jensen F.L. (1978). Grapevine response to concentrate and dilute application of two zinc compounds. Am. J. Enol. Vitic..

[60] Christensen L.P., Kasimatis A.N., Jensen F.L. (1982). Grapevine Nutrition and Fertilization in the San Joaquin Valley.

[61] Christensen P. (1986). Additives don’t improve zinc uptake in grapevines. California Agriculture.

[62] Moyer M.M., Singer S.D., Davenport J.R., Hoheisel G.-A. (2018). Vineyard Nutrient Management in Washington State.

[63] Hewitt E.J. (1966). Sand and Water Culture Methods Used in the Study of Plant Nutrition.

[64] Moore D.P., Mortvedt J.J., Giordano P.M., Lindsay W.L. (1972). Mechanisms of micronutrient uptake by plants. Micronutrients in Agriculture.

[65] Loneragan J.F., Nicholas D.J.D., Egan A.R. (1975). The availability and absorption of trace elements in soil-plant systems and their relation to movement and concentration of trace elements in plants. Trace Elements in Soil-Plant Animal Systems.

[66] Olsen S.R., Mortvedt J.J., Giordano P.M., Lindsay W.L. (1972). Micronutrient interactions. Micronutrients in Agriculture.

[67] Fageria V.D. (2001). Nutrient interactions in crop plant. J. Plant Nutr..

[68] Fageria N.K., Baligar V.C. (1999). Growth and nutrition concentrations of common bean, lowland rice, corn soybean, and wheat at different soil pH an an inceptisol. J. Pant Nutr..

[69] Shukla U.C., Mukhi A.K. (1980). Ameliorative role of Zn, K, and gypsum on maize: Growth under alkali soil conditions. Agron. J..

[70] Reich M., Shahbaz M., Prajapati D.H., Parmar S., Hawkesford M.J., De Kok L.J. (2016). Interactions of sulfate with other nutrients as revealed by H2S fumigation of chinese cabbage. Front. Plant Sci..

[71] Saeed M., Fox R.I. (1979). Influence of phosphate fertilization on zinc adsorption by tropical soils. Soil Sci. Soc. Am. J..

[72] Mandal L.N. (1980). Influence of phosphorus and zinc application in the availability of zinc, copper, iron, manganese and phosphorus in waterlogged rice soils. Soil Sci..

[73] Haldar M., Mandal L.N. (1981). Effect of phosphorus and zinc on the growth and phosphorus, zinc, copper iron and manganese nutrition of rice. Plant Sci..

[74] Mandal B., Mandal L.N. (1990). Effect of phosphorus application on transformation of zinc fraction in soil and on the zinc nutrition of lowland rice. Plant Soil.

[75] Alloway B.J. (2004). Fundamental aspects. Zinc in Soils and Crop Nutrition.

[76] Bavaresco L., Gatti M., Fregoni M. (2010). Nutritional deficiencis. Methodologies and Results in Grapevine Research.

[77] Iyengar S.S., Martens D.C., Miller W.P. (1981). Distribution and plant availability of soil zinc fractions. Soil Sci. Soc. Am. J..

[78] Eichert T., Kurtz A., Steiner U., Goldbach H.E. (2008). Size exclusion limits and lateral heterogeneity of the stomatal foliar uptake pathway for aqueous solutes and water-suspended nanoparticles. Physiol. Plant..

[79] Fernández V., Brown P.H. (2013). From plant surface to plant metabolism: The uncertain fate of foliar-applied nutrients. Front. Plant Sci..

[80] Van Goor B.J., Wiersma D. (1976). Chemical form of manganese and zinc in phloem exudates. Physiol. Plant..

[81] Tiffin L.O. (1977). The form and distribution of metals in plants: An overview. Proceedings of the Hanford Life Sciences Symposium.

[82] Loneragan J.F., Snowgall K., Robson A.D., Wardlaw I.F., Passioura J.B. (1976). Remobilization of nutrients and its significance in plant nutrition. Transport and Transfer Process in Plants.

[83] Marschner H. (1995). Mineral Nutrition of Higher Plants.

[84] Zhang Q., Brown P.H. (1999). Distribution and transport of foliar applied zinc in pistachio. J. Am. Soc. Hortic. Sci..

[85] Zhu J., Li J., Shen Y., Liu S., Zeng N., Zhan X., White J.C., Gardea-Torresdey J., Xing B. (2020). Mechanism of zinc oxide nanoparticle entry into wheat seedling leaves. Environ. Sci. Nano.

[86] Zou C.Q., Zhang Y.Q., Rashid A., Ram H., Savasli E., Arisoy R.Z., Ortiz-Monasterio I., Simunji S., Wang Z.H., Sohu V. (2012). Biofortification of wheat with zinc through zinc fertilization in seven countries. Plant Soil.

[87] Wang J.H., Mao H., Zhao H., Huang D., Wang Z. (2012). Different increases in maize and wheat grain zinc concentrations caused by soil and foliar applications of zinc in loess plateau, China. Field Crop. Res..

[88] Cruz T.N.M., Savassa S.M., Gomes M.H.F., Rodrigues E.S., Duran N.M., Almeida E., Martinelli A.P., Carvalho H.W.P. (2017). Shedding light on the mechanisms of absorption and transport of ZnO nanoparticles by plants via in vivo X-ray spectroscopy. Environ. Sci. Nano.

[89] Prasad T.N.V.K.V., Sudhakar P., Sreenivasulu Y., Latha P., Munaswamy V., Reddy K.R., Sreeprasad T.S., Sajanlal P.R., Pradeep T. (2012). Effect of nanoscale zinc oxide particles on the germination, growth and yield of peanut. J. Plant Nutr..

[90] Subbaiah L.V., Prasad T.N.V.K.V., Krishna T.G., Sudhakar P., Reddy B.R., Pradeep T. (2016). Novel effects of nanoparticulate delivery of zinc on growth, productivity, and zinc biofortification in maize (*Zea mays* L.). J. Agric. Food Chem..

[91] Zhang T., Sun H., Lv Z., Cui L., Mao H., Kopittke P.M. (2018). Using synchrotron based approaches to examine the foliar application of ZnSO_4_ and ZnO nanoparticles for field-grown winter wheat. J. Agric. Food Chem..

[92] Rossi L., Fedenia L.N., Sharifan H., Ma X., Lombardini L. (2019). Effects of foliar application of zinc sulfate and zinc nanoparticles in coffee (*Coffea arabica* L.) plants. Plant Physiol. Biochem..

[93] Kanellis A.K., Seymour G.B., Taylor J.E., Tucker G.A. (1993). Grape. Biochemistry of Fruit Ripening.

[94] Magalhães N. (2008). Tratado de Viticultura—A Videira, a Vinha e o “Terroir”.

[95] Chang E.H., Jung S.M., Hur Y.Y. (2014). Changes in the aromatic composition of grape cv. Cheongsoo wine depending on the degree of grape ripening. Food Sci. Biotechnol..

[96] Trad M., Boge M., Hamda H.B., Renard C.M.G.C., Harbi M. (2017). The Glucose-Fructose ratio of wild Tunisian grapes. Cogent Food Agric..

[97] Liang Z., Sang M., Fan P., Wu B., Wang L., Yang S., Li S. (2011). CIELAB coordinates in response to berry skin anthocyanins and their composition in Vitis. J. Food Sci..

[98] Regulation EU nº 251/2014 of the European Parliament and of the Council, 2014. https://eur-lex.europa.eu/homepage.html.

[99] Roohani N., Hurrell R., Kelishadi R., Schulin R. (2013). Zinc and its importance for human health: An integrative review. J. Res. Med. Sci..

[100] Direcção Geral de Agricultura Desenvolvimento Rural (1972). Carta de Capacidade de Uso do Solo de Portugal—Bases e Normas Adoptadas na Sua Elaboração.

[101] Pessoa M.F., Scotti-Campos P., Pais I., Feteiro A., Canuto D., Simões M., Pelica J., Pataco I., Ribeiro V., Reboredo F.H. (2016). Nutritional profile of the Portuguese cabbage (*Brassica oleracea* L var. costata) and its relationship with the elemental soil analysis. Emir. J. Food Agric..

[102] Pelica J., Barbosa S., Lidon F., Pessoa M.F., Reboredo F., Calvão T. (2018). The paradigm of high concentration of metals of natural or anthropogenic origin in soils—The case of Neves-Corvo mine area (Southern Portugal). J. Geochem. Explor..

[103] Rodier J., Legube B., Merlet N. (2009). L’Analyse de l’Eau.

[104] Piper A.M. (1944). A graphic procedure in the geochemical interpretation of water analyses. EOS Trans. Am. Geophys. Union.

[105] Rodrigues W.P., Martins M.Q., Fortunato A.S., Rodrigues A.P., Semedo J.N., Simões-Costa M.C., Pais I.P., Leitão A.E., Colwell F., Goulão L. (2016). Long-term air [CO_2_] strenghtens photosymthetic functioning and mitigates the impact of supra-optimal temperatures in tropical *Coffea arabica* and *C. canephora* species. Glob. Change Biol..

[106] Mangueze A.V.J., Pessoa M.F.G., Silva M.J., Ndayiragije A., Magaia H.E., Cossa V.S.I., Reboredo F.H., Carvalho M.L., Santos J.P., Guerra M. (2018). Simultaneous zinc and selenium biofortification in rice. Accumulation, localization and implications on the overall mineral content of the flour. J. Cereal Sci..

[107] Reboredo F.H.S., Ribeiro C.A.G. (1984). Vertical distribution of Al, Cu, Fe and Zn in soil salt marshes of the Sado estuary, Portugal. Int.J. Environ. Stud..

[108] Cardoso P., Mateus T.C., Velu G., Singh R.P., Santos J.P., Carvalho M.L., Lourenço V.M., Lidon F., Reboredo F., Guerra M. (2018). Localization and distribution of Zn and Fe in grains of biofortified bread wheat lines through micro- and triaxial-X-ray fluorescence spectrometry. Spectrochim. Acta Part B At. Spectrosc..

