# Supplementary material for: Enrichment of Grapes with Zinc-Efficiency of Foliar Fertilization with ZnSO4 and ZnO and Implications on Winemaking
Source: Plants (Basel). 2022 May 25;11(11):1399. doi: 10.3390/plants11111399 (PMC9182840; doi:10.3390/plants11111399)
Supplement: Supplementary file 1 [file plants-11-01399-s001.zip › plants-1714867-supplementary.pdf]

Varieties	Treatments						
	Control	ZnO (150 g ha^{-1})	ZnO (450 g ha^{-1})	ZnO (900 g ha^{-1})	$ZnSO_4$ (150 g ha^{-1})	$ZnSO_4$ (450 g ha^{-1})	$ZnSO_4$ (900 g ha^{-1})
**Castelão**	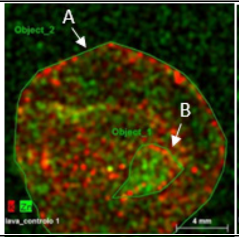	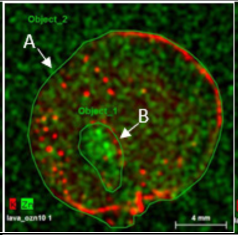	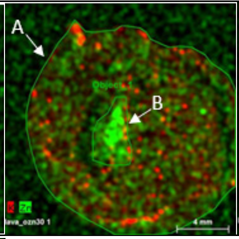	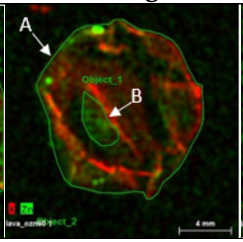	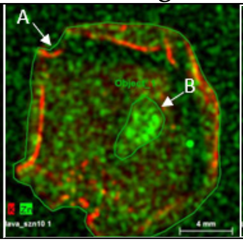	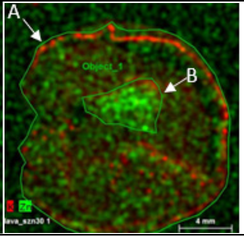	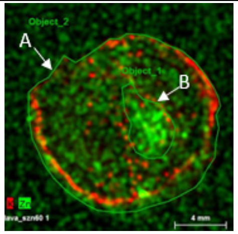
**Moscatel**	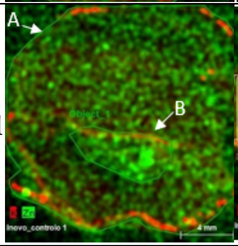	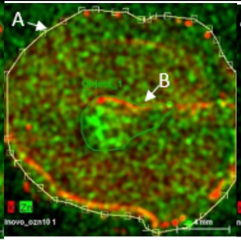	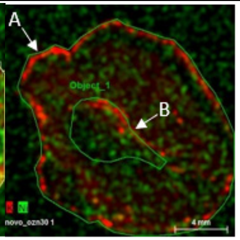	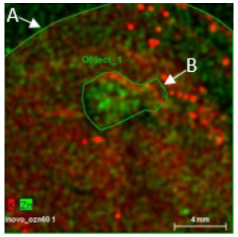	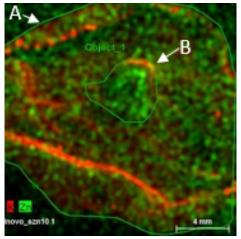	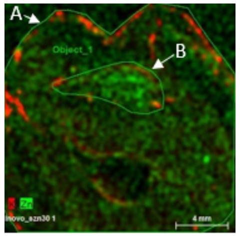	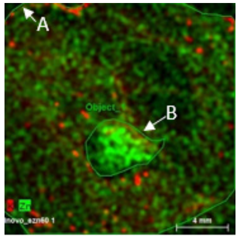

Figure S1. Identification of skin (A) and seed (B) regions in grapes of *Vitis vinifera* varieties Castelão and Moscatel, where Zn content was measured with the µ-EDXRF system (M4 Tornado™, Bruker, Germany).

Table S1. Average ± SE ($n = 3$) of dry weight and total soluble solids (expressed as °Brix) in grapes of *Vitis vinifera* varieties Castelão and Moscatel. Letters a, b indicate significant differences among treatments in each variety, whereas letters A and B indicate the significant differences between varieties in each treatment (statistical analysis using the single-factor ANOVA test, $p \leq 0.05$).

Treatments	Castelão	Moscatel
	Dry Weight (%)	
Control	20.10 ± 0.39aA	15.91 ± 1.17aA
ZnO (150 g ha^{-1})	21.67 ± 2.28aA	21.84 ± 0.76aA
ZnO (450 g ha^{-1})	22.22 ± 2.10aA	19.11 ± 1.56aA
ZnO (900 g ha^{-1})	21.78 ± 1.26aA	19.04 ± 2.36aA
$ZnSO_4$ (150 g ha^{-1})	20.08 ± 0.57aA	21.33 ± 3.90aA
$ZnSO_4$ (450 g ha^{-1})	23.22 ± 0.25aA	19.40 ± 4.68aA
$ZnSO_4$ (900 g ha^{-1})	21.35 ± 1.33aA	20.68 ± 2.05aA
	°Brix—%TSS	
Control	18.00 ± 0.05aA	14.47 ± 0.07bB
ZnO (150 g ha^{-1})	17.67 ± 1.15aA	21.20 ± 1.62aA
ZnO (450 g ha^{-1})	19.77 ± 0.32aA	15.93 ± 0.76a,bB
ZnO (900 g ha^{-1})	19.87 ± 0.58aA	16.03 ± 0.84a,bB
$ZnSO_4$ (150 g ha^{-1})	19.30 ± 0.85aA	18.40 ± 0.70a,bA
$ZnSO_4$ (450 g ha^{-1})	19.63 ± 0.24aA	14.50 ± 0.24bB
$ZnSO_4$ (900 g ha^{-1})	19.03 ± 0.50aA	17.33 ± 1.52a,bA

Table S2. Average ± SE ($n = 3$) of colorimeter parameters of the skin of grapes of *Vitis vinifera* varieties Castelão and Moscatel. Letters a, b indicate significant differences among treatments in each variety, whereas letters A and B indicate the significant differences between each parameter for both varieties in each treatment (statistical analysis using the single-factor ANOVA test, $p \leq 0.05$).

Treatments	Colorimetric Parameters					
	Castelão			Moscatel		
	L *	a *	b *	L *	a *	b *
Control	19.69 ± 1.83aA	-0.32 ± 0.09aA	-0.68 ± 0.15aB	20.04 ± 4.97aA	-3.41 ± 0.40a,bB	3.16 ± 0.20aA
ZnO (150 g ha^{-1})	17.60 ± 4.21aA	-0.65 ± 0.07aA	-1.22 ± 0.82aB	25.65 ± 5.26aA	-1.25 ± 0.80aA	5.04 ± 0.57aA
ZnO (450 g ha^{-1})	23.46 ± 1.74aB	-0.44 ± 0.15aA	-1.98 ± 0.57aB	32.86 ± 1.51aA	-3.66 ± 0.62a,bB	5.00 ± 0.12aA
ZnO (900 g ha^{-1})	19.13 ± 1.47aA	-0.54 ± 0.13aA	-1.17 ± 0.38aB	27.80 ± 3.42aA	-2.98 ± 0.48a,bB	5.01 ± 0.74aA
$ZnSO_4$ (150 g ha^{-1})	20.56 ± 0.64aA	-0.20 ± 0.28aA	-1.74 ± 0.33aB	27.41 ± 3.56aA	-3.84 ± 0.30a,bB	3.25 ± 0.24aA
$ZnSO_4$ (450 g ha^{-1})	16.78 ± 1.24aB	-0.64 ± 0.05aA	-1.03 ± 0.51aB	32.28 ± 0.35aA	-4.11 ± 0.79bB	5.03 ± 0.63aA
$ZnSO_4$ (900 g ha^{-1})	15.93 ± 1.56aB	-0.46 ± 0.12aA	-0.51 ± 0.15aB	30.35 ± 2.08aA	-2.67 ± 0.19a,bB	3.09 ± 0.37aA
